# Septochoanal polyp: a case report and literature review

**DOI:** 10.1097/RC9.0000000000000850

**Published:** 2026-08-12

**Authors:** Ahmad Alkheder, Hala Alibrahim, Diana Saleem, Ghoufran Ibraheem Najeeb, Adel Azar, Adham Bader Aldeen Mohsen

**Affiliations:** a Department of Otorhinolaryngology, Al-Mouwasat University Hospital, Damascus University, Damascus, Syria; b Faculty of Medicine, Damascus University, Damascus, Syria; c Faculty of Medicine, Syrian Private University, Damascus, Syria; d Department of Pathology and Cytopathology, Al Mouwasat University Hospital, Damascus, Syria; e Faculty of Medicine, Homs University, Homs, Syria

**Keywords:** choanal polyps, diagnosis, pathogenesis, review, septochoanal polyp, treatment

## Abstract

**Introduction and importance:**

The septochoanal polyp is an exceptionally rare variant of a choanal polyp, distinguished by its origin from the nasal septum rather than the paranasal sinuses. To our knowledge, fewer than fifteen cases of true septochoanal polyps have been reported to date.

**Presentation of the case:**

A 65-year-old male presented with persistent right-sided nasal obstruction and hyponasality despite prior septal surgery. Nasal endoscopy revealed a large anterior septal perforation and a substantial polypoid lesion extending into the nasopharynx. Computed tomography confirmed a homogeneous mass originating from the superior nasal septum, with no sinus involvement. The patient underwent successful, complete endoscopic excision of the lesion, which was delivered transorally because of its size.

**Clinical discussion:**

This case contributes to the scarce literature on this rare entity. The patient’s history of septal surgery and the subsequent perforation support the theory that mechanical stress and turbulent nasal airflow may be significant factors in the pathogenesis of septal-origin polyps. Endoscopic surgery, entailing complete removal of the polyp along with its pedicle and a small mucosal margin, is the definitive treatment, resulting in a low recurrence rate and an excellent prognosis.

**Conclusion:**

The septochoanal polyp, while rare, should be considered in the differential diagnosis of a unilateral choanal mass. A combination of endoscopic examination and computed tomography is essential for precise preoperative localization of its septal origin. Endoscopic surgical excision remains the treatment of choice, offering a definitive cure. This case further highlights the potential role of disrupted nasal aerodynamics in the development of this unusual polyp.

## Introduction

Choanal polyps are distinct nasal polyps with a single stalk and posterior growth toward the choana. Most of these lesions, antrochoanal polyps, originate from the paranasal sinus mucosa, usually the maxillary sinus^[^1^]^. In contrast, a choanal polyp from the nasal septum (termed a septochoanal polyp) is exceptionally rare^[^1^]^. Since the first description of a choanal polyp of septal origin, few cases have been reported in the global literature. To our knowledge, fewer than fifteen true septochoanal polyps have been reported. These polyps typically arise from the superior or posterior nasal septum and extend into the nasopharynx, often causing unilateral nasal obstruction, hyponasality, or snoring^[^1–4^]^. Their pathogenesis is uncertain, but inflammatory processes, allergic factors, and mechanical stress from altered nasal airflow due to septal deviation have been proposed as potential contributing mechanisms^[^2,5^]^. Preoperative identification of septal origin is crucial because it influences surgical planning and distinguishes this benign inflammatory lesion from other nasal and nasopharyngeal neoplasms, such as inverted papilloma, respiratory epithelial adenomatoid hamartoma (REAH), or juvenile angiofibroma^[^2,3,6^]^. The standard curative treatment is complete endoscopic excision of the polyp, its pedicle, and a small margin of surrounding healthy mucosa to prevent recurrence^[^3,4,7^]^. This report describes a large septochoanal polyp in a patient with prior septal surgery, adding to the limited literature on this rare condition and highlighting key diagnostic and management considerations. This work is reported in line with the Surgical CAse REport 2025 criteria.

HIGHLIGHTS

- A rare septochoanal polyp from the nasal septum adds to the scarce literature.
- Prior septal surgery with perforation alters nasal airflow, a key factor in pathogenesis.
- Preoperative identification of septal attachment by endoscopy and/or CT is crucial for the differential diagnosis.
- Endoscopic excision, including the pedicle and margins, is definitive and confers an excellent prognosis.
- A septochoanal polyp should be considered in any patient with a unilateral choanal mass.

## Case presentation

A 65-year-old male patient presented with right-sided nasal obstruction, hyponasality, and a sensation of post-nasal drip. The patient reported that 5 months earlier, he had undergone surgery to correct a deviated nasal septum, which had been previously diagnosed by another physician. He noted partial improvement in nasal obstruction after the surgery, but his nasal tone showed little to no improvement. The patient had hypertension and type 2 diabetes mellitus. He had a history of allergic rhinitis, but the treating physician considered septal deviation to be the primary cause of nasal obstruction; therefore, he was not on regular anti-allergic medications and underwent septoplasty. He was a current smoker.

**Figure 1.**
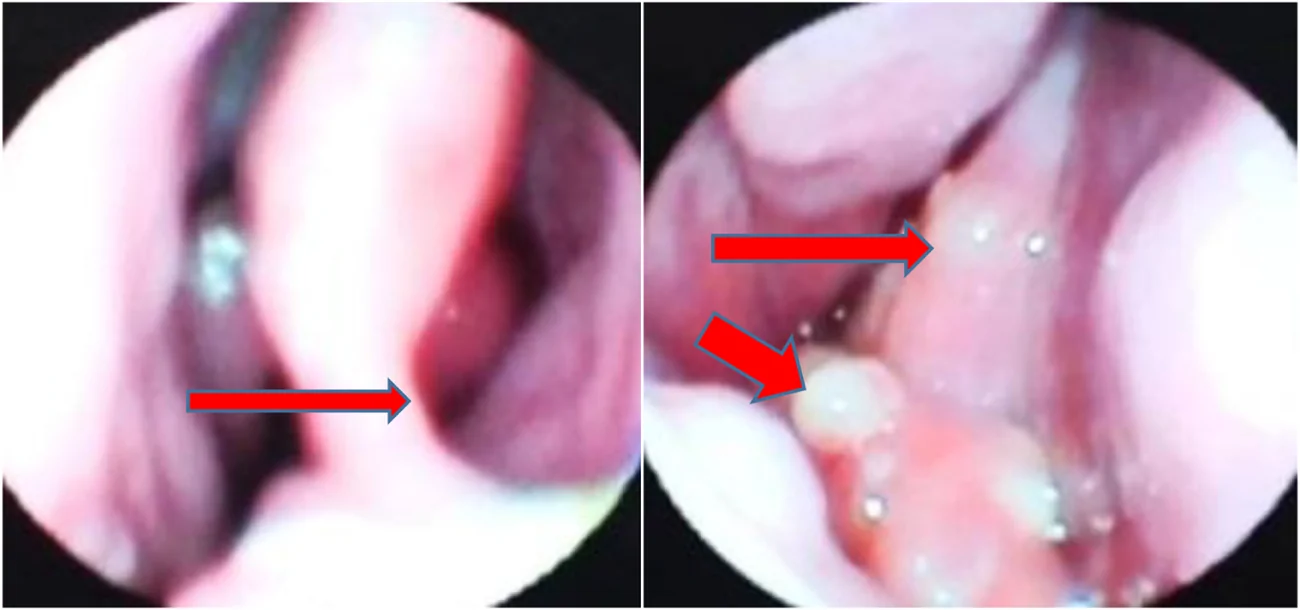
The left image depicts a large perforation of the anterior septum. The right image shows a sizable lesion on the floor of the right nasal cavity.

**Figure 2.**
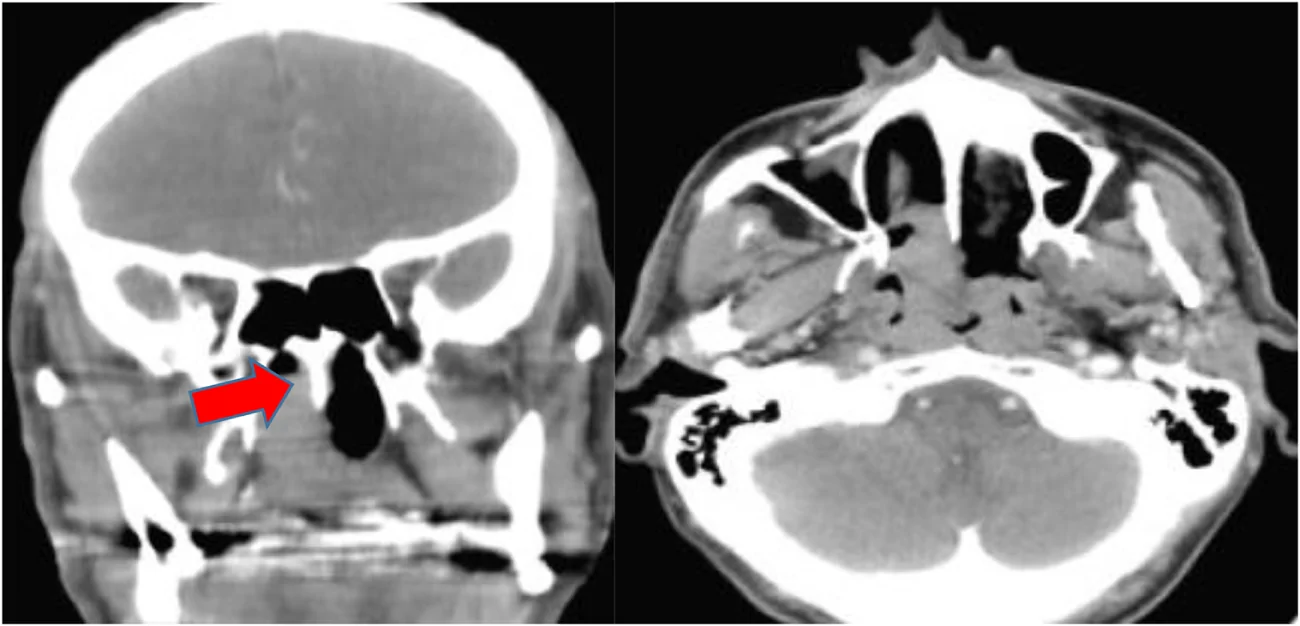
Contrast-enhanced coronal and axial CT images showing a homogeneous, intermediate-density lesion extending into the nasal floor and reaching the nasopharynx.

Fiber-optic nasal endoscopy revealed a large anterior perforation in the nasal septum (Fig. 1, left) and a large lesion on the right side extending down to the nasal floor, with a pedicle originating from the posterior-superior part of the nasal cavity, although its exact origin could not be determined. The lesion extended to the nasal floor and reached the nasopharynx (Fig. 1, right). Contrast-enhanced CT scan showed a homogeneous, intermediate-density lesion originating from the superior part of the nasal septum, extending to the nasal floor and nasopharynx. The lesion measured approximately 5 cm in its largest dimension. No bony erosion, destruction, or remodeling of the nasal septum, skull base, or maxillary sinuses was observed. The maxillary, sphenoid, and ethmoid sinuses were normal (Fig. 2).

**Figure 3.**
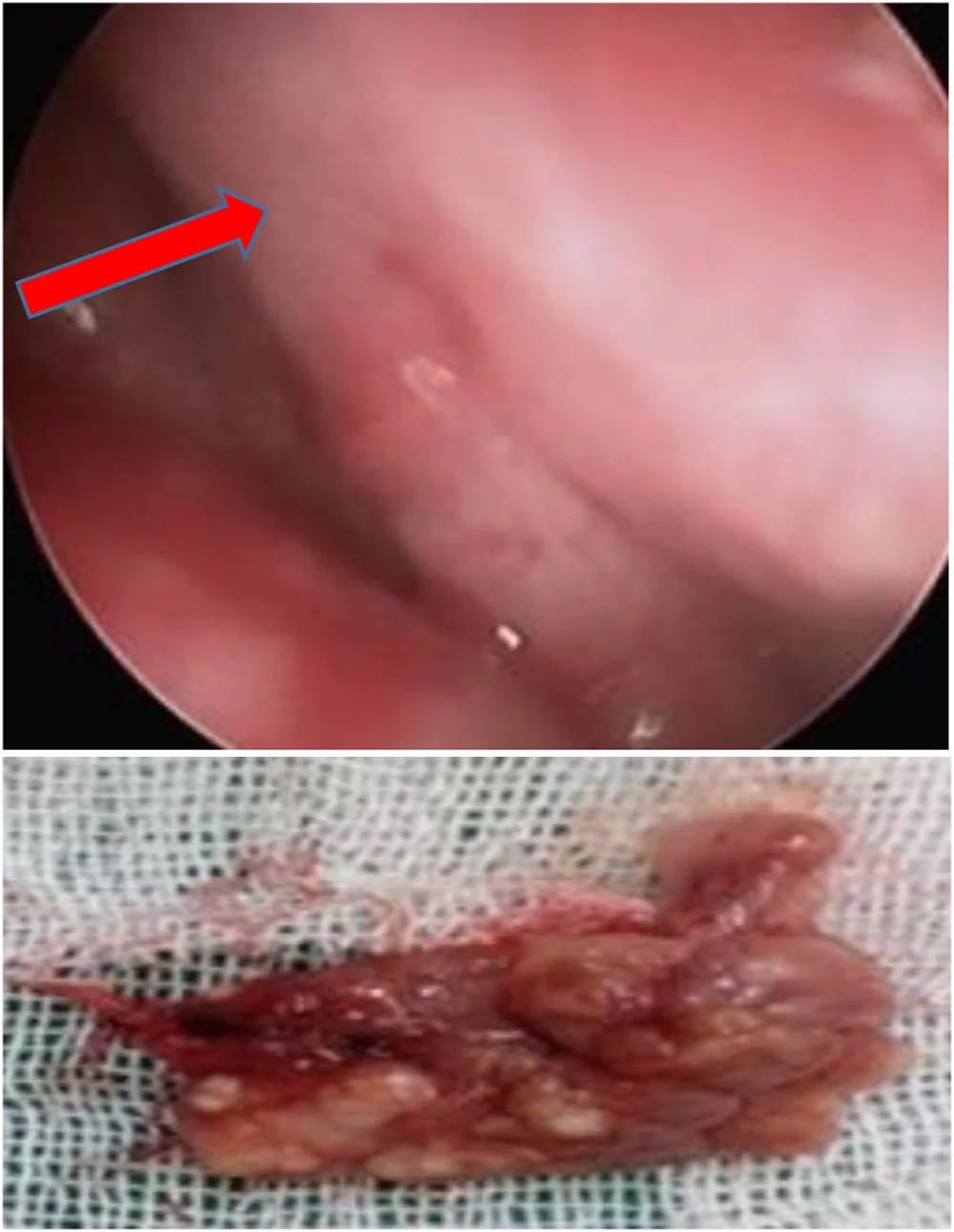
At the top, an endoscopic view during the procedure illustrates the stalk attached to the posterior-superior aspect of the septum. Below, the lesion after excision measures 5 × 3.5 × 3 cm.

Functional endoscopic sinus surgery was performed under general anesthesia. The patient was positioned supine with the head elevated by 15 degrees and slightly turned toward the surgeon, following standard endoscopic sinus surgery protocol. The lesion was exposed, and the pedicle was mechanically transected from the nasal septum with cup forceps. The pedicle attachment site was then cauterized with bipolar cautery to prevent recurrence. Because of its large size, the lesion was pushed into the oropharynx and removed through the mouth (Fig. 3).

The patient was discharged on the same day in good general condition. Histopathological examination revealed an allergic polyp with no evidence of fungi or malignant changes (Supplemental Digital Content Figure, available at: https://links.lww.com/IJSCR/A113). The patient showed marked improvement in symptoms, with complete resolution of hyponasality. Postoperatively, the patient was followed up for 4 months. He reported complete resolution of symptoms with no evidence of recurrence. Further endoscopic examination was not possible because the patient had traveled abroad. However, he was instructed that if any symptoms recurred, he should seek medical attention either at our center or at any local hospital in the country where he resides.

## Discussion

The septochoanal polyp represents an exceptionally rare clinical entity within the spectrum of choanal polyps, with our case contributing to the limited body of literature documenting this condition. Since the initial description by Bailey, very few true septochoanal polyps have been reported, underscoring its rarity^[^1,4^]^. The pathogenesis of these polyps remains incompletely understood, but several theories have been proposed. Inflammatory processes and allergic factors are commonly implicated in the development of nasal polyps in general^[^1,2^]^. However, the role of mechanical stress and altered nasal airflow dynamics may be particularly relevant for septal-origin polyps^[^2,3^]^.

Our patient’s history of prior septal surgery adds a noteworthy dimension to the discussion. Nasal septal deviation has been suggested to alter the intranasal air stream, with the concave side experiencing stronger airflow^[^2^]^. This increased airflow and subsequent wall shear stress on the concave-side mucosa could potentially serve as a chronic irritant, triggering inflammation and polyp formation^[^2,3^]^. Our case, where the polyp was found on the side of a significant anterior septal perforation-a condition that drastically alters normal nasal aerodynamics-lends support to the theory that mechanical factors and turbulent airflow may contribute to the localized mucosal response leading to polypogenesis^[^2,3^]^; given the current limited evidence, this interpretation remains hypothesis-generating rather than inferential, and further studies are needed to establish a mechanistic link. This aligns with the observation by Numano *et al*, who also reported a septochoanal polyp on the concave side of a deviated nasal septum^[^2^]^.

Preoperative identification of the polyp’s septal origin is paramount for accurate diagnosis and surgical planning^[^1,3,4^]^. As demonstrated in our case and others, nasal endoscopy is the cornerstone for visualizing the pedicle attachment to the posterior or superior septum^[^1,3,4,7^]^. Computed tomography (CT) plays a complementary role, confirming the absence of sinus involvement – a key feature distinguishing it from the more common antrochoanal polyp – and precisely delineating the extent of the lesion^[^1–3,5^]^. In the present case, several differential diagnoses were considered and systematically excluded based on the available clinical, radiological, and histopathological findings. Inverted papilloma typically presents with a granular, papillary surface on endoscopy and may show focal hyperostosis or bone remodeling on CT; our patient’s lesion was smooth, translucent, and non-papillary, with no bony changes. Juvenile angiofibroma, which occurs almost exclusively in adolescent males, demonstrates intense contrast enhancement and a characteristic anterior extension to the sphenopalatine foramen on imaging; our patient was 65 years old, and CT showed a homogeneous, non-enhancing mass with no vascular blush. REAH arises mainly from the olfactory clefts or superior septum and has a glandular pattern on histology; our lesion originated from the superior septum but showed no glandular hyperplasia, only edematous, allergic-type stroma. Meningoencephalocele was excluded by the absence of any skull base defect or intracranial connection on contrast-enhanced CT. Other malignant sinonasal tumors were deemed unlikely given the lack of irregular mucosal thickening, bony erosion, or heterogeneous enhancement. Thus, the diagnosis of a benign septochoanal polyp was confirmed. Therefore, a high index of suspicion is necessary to differentiate this benign inflammatory polyp from more aggressive pathologies^[^1,2^]^.

The standard curative treatment for a septochoanal polyp is complete endoscopic surgical excision^[^1,3,4^]^. The surgical principle emphasizes removal of the entire polyp along with its pedicle and a small portion of the surrounding healthy septal mucosa to minimize the risk of recurrence^[^1,3–5^]^. In our case, the large size of the polyp necessitated delivery through the oropharynx, as described by Birkent *et al* and Cho *et al*, a technique that is feasible and effective for such substantial lesions^[^1,4^]^. The endoscopic approach offers excellent visualization, facilitates precise removal of its base, and is associated with minimal morbidity^[^1,3,4^]^. The reported recurrence rate for septochoanal polyps appears to be very low, likely due to the ease of identifying and completely resecting the pedicle from its accessible septal location, contrasting with the higher recurrence rates sometimes seen with antrochoanal polyps^[^1–3^]^.

Histopathological examination invariably confirms the inflammatory nature of the polyp, typically showing edematous stroma, mucous glands, infiltration by chronic inflammatory cells such as lymphocytes and plasma cells, and a variable presence of eosinophils^[^1,2,5^]^. Our patient’s histology was consistent with an allergic polyp. While rare variants with metaplastic ossification have been reported, these are exceptional findings that can pose a diagnostic challenge on imaging, but do not alter the fundamental benign nature or management strategy of the lesion^[^5,6^]^.

A key strength of this report is the detailed clinical and functional characterization of a rare presentation. However, several limitations should be acknowledged. The absence of long-term follow-up means we cannot comment on disease trajectory or treatment durability. Additionally, the lack of objective airflow analysis (e.g., spirometry or peak flow measurements) restricts the physiological interpretation of the reported symptoms.

## Conclusion

The septochoanal polyp is an exceptionally rare benign entity that should be considered in the differential diagnosis of a unilateral choanal mass. Precise preoperative localization of its septal origin via endoscopy and CT is crucial for distinguishing it from other pathologies and for guiding management. Endoscopic surgical excision, including the pedicle and a small mucosal margin, remains the definitive treatment.

## Data Availability

All data are available from the corresponding author on reasonable request. The case has not been presented at a conference or regional meeting.
